# The Spirit Form

**Published:** 1887-09

**Authors:** 


### THE SPIRIT FORM.

Many go about this earth as though their outer bodies, and the garments that clothe them, were the only important things in life. Most of them have a vague idea that they shall live again somewhere in space, but it is a preposterous thing to them to suppose they will at all resemble their present selves. They speculate upon the question of the soul's appearance, presuming on all shapes but their own. And, indeed, we do not doubt but that our spiritual forms will correspond very nearly to the lives we live in the body. Thoughts and deeds mould the face, why not the soul? But this does not occur to them. They look on the soul as a possible remainder, something left over from the life of the body that is to go idling through all eternity, aimless and senseless. The emblems in nature of life, death and resurrection have no meaning, and even the revered Book that positively assures them of each, does not seem to awaken their minds to the truth of the spiritual about them. Nothing is so obdurate as the human mind when blinded by groundless prejudice. Soon, the finger of death is laid upon the pulse, and the cramped soul goes out with a wail into the darkness, dense with ignorance.—*Golden Gate.*

